# Heterogeneous groups overcome the diffusion of responsibility problem in social norm enforcement

**DOI:** 10.1371/journal.pone.0208129

**Published:** 2018-11-30

**Authors:** Wojtek Przepiorka, Andreas Diekmann

**Affiliations:** 1 Department of Sociology/ICS, Utrecht University, Utrecht, Netherlands; 2 Department of Humanities, Social and Political Sciences, ETH Zurich, Zurich, Switzerland; 3 Institute of Advanced Studies, Berlin, Germany; Middlesex University, UNITED KINGDOM

## Abstract

Social norms promote cooperation in everyday life because many people are willing to negatively sanction norm breakers at a cost to themselves. However, a norm violation may persist if only one person is required to sanction the norm breaker and everyone expects someone else to do it. Here we employ the volunteer’s dilemma game (VOD) to model this diffusion of responsibility in social norm enforcement. The symmetric VOD is a binary choice game in which all actors have the same costs of and benefits from cooperation and only one actor’s cooperation is required to provide the collective good for the group. The asymmetric VOD differs from the symmetric VOD in one (strong) actor having lower costs of cooperation. In a laboratory experiment, we find that, in line with the diffusion of responsibility hypothesis, subjects’ propensities to sanction the norm breaker decrease with group size in the symmetric VOD. In the asymmetric VOD, groups tacitly coordinate on the strong subject to sanction the norm breaker alone. Although at first lower in larger groups, strong subjects’ sanctioning rates increase over time and reach equally high levels across different group sizes. Our results show that heterogeneous groups can be more effective in achieving norm compliance than groups of all equals because they naturally evade diffusion of responsibility in social norm enforcement.

## Introduction

Cooperation problems studied by biologists, economists and other social scientists are often non-linear in nature [1,2,3,4,5]. In game theory, step-level collective goods games account for a specific type of non-linearity in the production function of collective goods [6,7,8,9]. The volunteers dilemma (VOD) is a prominent example. The VOD is a binary choice game in which a single actor’s cooperation is necessary and sufficient to provide the collective good. Cooperators and free riders alike benefit from the collective good, but if everybody defects, no one receives anything [10]. The VOD and its extensions have been applied to a large number of collective good problems in such diverse fields as behavioural economics [11], biology [12], computer science [13,14], sociology [15,16,17], and anthropology [18]. Here we show how the VOD can be applied to study diffusion of responsibility in social norm enforcement.

Social psychologists have extensively studied the causes of bystander intervention in emergency situations and diffusion of responsibility (aka bystander effect) has been of particular interest in this strand of research [19,20]. The diffusion of responsibility is a mechanism explaining the decreasing inclination of bystanders to help in an emergency situation the more other bystanders are present. In other words, diffusion of responsibility describes a group size effect by which the likelihood that the collective good is produced decreases with group size [10]. Research on social dilemmas [21,22,23] has produced a vast body of literature that investigates the effect of group size on cooperation and the production of collective goods [24,25,26,27,28]. Although the evidence is mixed, the literature is less equivocal in its concluding that the production function of a collective good is a major factor driving the group size effect.

Since Darley and Latané [19] corroborated the diffusion of responsibility effect empirically five decades ago, numerous studies have replicated it in various fields of application. Research has established several mechanisms involved in helping and other type of volunteering situations [29] and has explored various characteristics attenuating or strengthening diffusion of responsibility (for systematic reviews see [20,30].

Several authors proposed general models describing and explaining diffusion of responsibility. For example, Latané [31] (see also [32]) proposed an inverse power function to describe the group size effect while Piliavin et al. [33] suggested modelling the helping decision by a cost-benefit model. Although valuable steps forward, both these and other modelling efforts largely neglected the strategic nature of volunteering situations and game theoretic approaches to the modelling of the diffusion of responsibility effect. In what follows, we bring together psychology and game theory by modelling the volunteering situation as VOD and show how small variations in actors’ game payoffs can lead to an *endogenous* solution of the diffusion of responsibility problem. We argue and show that this solution can be applied to the diffusion of responsibility problem in social norm enforcement.

## The model

**Fig 1:**
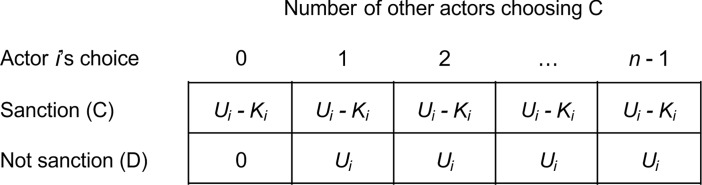
The volunteer’s dilemma (VOD) game from actor *i*’s perspective. In the VOD, actor *i* chooses between sanctioning (C) and declining to sanction (D). If *i* chooses C, *i* incurs a cost *K*_*i*_ but earns *U*_*i*_ as the collective good is produced. If *i* chooses D, *i*’s payoff depends on what the other actors do. If all other actors choose D, the collective good is not produced and no one earns anything. If at least one of the other actors chooses C, the collective good is produced and *i* earns *U*_*i*_. Since in a VOD *U*_*i*_ > *K*_*i*_ > 0, choosing D is not a dominant strategy, yet all actors prefer another actor to choose C while they choose D.

The basic VOD is a binary choice, symmetric *n*-person game in which a single actor’s cooperation is sufficient to provide the collective good *U* at a cost *K*. Cooperators and free riders alike benefit from the collective good. However, if everybody free rides, no one receives anything [10] (Fig 1).

In the symmetric VOD, the costs and benefits from choosing C are the same for all actors (i.e. *U*_*i*_ = *U*_*j*_ and *K*_*i*_ = *K*_*j*_ ∀ *i* ≠ *j*). The symmetric VOD has *n* asymmetric pure strategy Nash equilibria, in which one actor chooses C and all other actors choose D. A pure strategy equilibrium is, however, difficult to coordinate on tacitly in a one-shot game. The symmetric VOD also has a symmetric mixed strategy equilibrium, in which all actors have the same probability to choose C: $p_{i}^{*}=1-\sqrt[{n-1}]{K_{i}/U_{i}}$ [9]. The unique symmetric Nash equilibrium implies a monotonically decreasing function of the probability of cooperation (*p*) with group size (*n*). We call this property of the symmetric VOD the “diffusion of responsibility” or “bystander” effect [19] (see also [34]). A slight variation of the model allows for individual heterogeneity, i.e. cooperation costs (*K*_*i*_) or benefits (*U*_*i*_) vary across actors. The resulting asymmetric VOD has very different strategic properties.

In the asymmetric VOD with one “strong” actor *i* and *n*– 1 “weak” actors *j*, *U*_*i*_ > *U*_*j*_ and/or *K*_*i*_ < *K*_*j*_ for all *j* ≠ *i*. In the asymmetric game, the mixed strategy equilibrium probability of cooperation is: $p_{i}^{*}=1-\frac{U_{i}}{K_{i}}{\left[\prod_{j=1}^{n}\frac{K_{j}}{U_{j}}\right]}^{\left(\frac{1}{n-1}\right)}$. Paradoxically, this equilibrium implies that the strong actor chooses C with the lowest probability [35]. The mixed strategy equilibrium is, however, not evolutionary stable. The evolutionary stable equilibrium is the strategy profile in which the strong player cooperates while all other players defect, irrespective of group size. This asymmetric, pure strategy equilibrium is also selected by the Harsanyi-Selten rationality theory [36].

Axioms of strategic rationality imply the selection of the mixed Nash equilibrium in the symmetric VOD. In contrast, in the asymmetric game a unique asymmetric equilibrium is selected where the “strongest” player (the actor with the largest difference between *U* and *K*) cooperates and all other, “weaker” actors defect [35,36]. Experiments on strategic human behaviour support these predictions [37], and analyses of replicator dynamics demonstrate that the strategy profile “strongest actor cooperates / weaker actors defect” constitutes an evolutionary stable equilibrium [38] (see the online Supporting Information, S1 Additional study material and analyses).

The game theoretic analysis of the symmetric and asymmetric VOD allows for an interesting and important insight. At least in theory, asymmetry solves the VOD because the strongest actor will always provide the collective good–independent of group size. As a consequence, diffusion of responsibility is only to be expected in the symmetric VOD, whereas it will be absent if the VOD is asymmetric. Note that this is different from solutions that rely on an established leader or status hierarchies [39,40,41,42]. Appointing a leader to produce the public good alone solves the VOD exogenously and presupposes the existence of a legitimate authority. Here we argue that arbitrary, observable differences in individuals’ costs of and/or benefits from producing the collective good can solve the VOD endogenously because group members tacitly coordinate on the strongest member to volunteer [15]. Note, moreover, that this mechanism may only be effective in solving step-level collective goods problems, in which the full contribution of a specific number of group members is required. In experiments with linear collective goods games, arbitrary differences in subjects’ endowments or payoffs have been shown to have counterproductive effects on the efficient production of these collective goods [43,44,45,46].

In what follows, we experimentally test the hypothesis that the diffusion of responsibility effect decreases as a consequence of the switch from the symmetric to the asymmetric VOD. Cramer et al. [47] were the first to observe this effect in a field experiment, in which they varied group size (*n* = 1 vs *n* = 2) and competence of the bystander (trained nurse vs student). They found that, in the presence of an observer, trained nurses were as likely to help when hearing someone falling from a ladder as subjects in the absence of an observer, whereas students’ likelihood of helping was lower. Here we study this effect by means of a computerized laboratory experiment. We use the game theoretic model of the symmetric and asymmetric VOD to derive predictions about subjects’ propensity to enforce a social norm. How social norms are enforced through sanctions has been of long-lasting interest to social scientists in general and sociologists in particular [48,49,50,51,52,53]. We argue and show that already relatively small differences in subjects’ sanctioning costs can substantially attenuate diffusion of responsibility in norm enforcement if only one person is necessary and sufficient to enforce the social norm (see also [54]).

## Methods

### Experimental game

We use the “stealing game” with a sanctioning option to emulate a situation in which a norm violation can be negatively sanctioned and this sanction can be subject to diffusion of responsibility [37,55]. The stealing game is a simple representation of a situation in which one actor has the possibility to benefit from breaking a social norm at a cost for others. For example, someone who enjoys listening to loud music in public transport benefits from breaking a social norm but, at the same time, “steals” utility from others by producing negative externalities [54,56]. In this situation the intervention of only one person is necessary to stop the norm breaker. Accordingly, in the stealing game an actor X has the opportunity to steal a certain amount (*U*) from each of *n* other group members. If X steals, he or she gains *nU*, but each of the *n* “victims” has the possibility to sanction the thief thereby reclaiming the stolen money. A sanction is a costly cooperative action (with costs *K*) and reclaims the stolen money (*U*) for all *n* victims, irrespective of whether they had sanctioned or not. We further introduce a penalty condition in which a thief is not only obliged to pay back the stolen money but also incurs a penalty, if at least one of the *n* victims sanctions. With *U* > *K* > 0, the *n* victims face a volunteer’s dilemma, irrespective of whether the thief can incur a penalty or not [8,37].

### Experimental design

In our experiment, we vary the number of potential victims (*n* = 2 vs *n* = 5) and the type of VOD game (symmetric vs asymmetric) in a 2 × 2 between-subject factorial design. Every subject makes decisions in 30 consecutive rounds of the stealing game with sanctioning option; first 15 rounds without and then 15 with a penalty threat for the thief. In each round, the role of X, the role of potential victims, and the role of strong and weak subjects in the asymmetric VOD are assigned randomly. We assign subjects’ roles randomly rather than having them assume the same role in every round for two reasons. First, experiencing the game in different roles allows subjects to better appreciate the strategic nature of the decision situation. Second, aggregate behaviour in particular roles is less dependent on the idiosyncrasies of the subjects assuming these roles. Moreover, we use stranger matching, i.e. after every round, groups are disbanded and randomly formed anew.

In every round, subject X first decides whether to steal 50 monetary units (MU) from each of the other *n* group members. If X steals, each victim decides independently whether or not to sanction to reclaim the stolen amount for the entire group (*U* = 50). Sanctioning costs are *K* = 25 for subjects in the symmetric VOD and for the strong subject in the asymmetric VOD, while sanctioning costs are higher (*K* = 35) for the weak subjects in the asymmetric VOD. In the second 15 rounds, a thief also incurs a penalty of 60 MU, if at least one of the victims sanctions. Hence, the second part of our experiment allows us to assess the impact of negative monetary sanctions on subjects’ behaviour in the stealing game. Previous research demonstrates that negative monetary sanctions can have a substantial, positive effect on cooperation in linear collective goods games [57,58,59]. Correspondingly, in our case, we expect the penalty to have a strong negative effect on the stealing rate. However, a low stealing rate will distort the conditions under which we can test our hypotheses on the diffusion of responsibility in norm enforcement. Therefore, we introduce the penalty only in the second part, i.e. after the 15 rounds without an additional penalty (also see [37]). Table 1 summarizes the parametrization of our experimental game and our experimental design.

**Table 1:** Experimental games and experimental design.

	symmetric		asymmetric	
	*n* = 2 *U* = 50 MU *K* = 25 MU	*n* = 5 *U* = 50 MU *K* = 25 MU	*n* = 2 *U* = 50 MU *K*_s_ = 25 MU *K*_w_ = 35 MU	*n* = 5 *U* = 50 MU *K*_s_ = 25 MU *K*_w_ = 35 MU
part 1: rounds 1–15	no penalty *P* = 0 MU	no penalty *P* = 0 MU	no penalty *P* = 0 MU	no penalty *P* = 0 MU
part 2: rounds 16–30	Penalty *P* = 60 MU	Penalty *P* = 60 MU	Penalty *P* = 60 MU	Penalty *P* = 60 MU

*n*: group size; *U*: Benefit from cooperation; *K*: cost of cooperation; *K*_s_: cost of cooperation for strong player; *K*_w_: cost of cooperation for weak player; *P*: penalty.

Our design does not allow us to disentangle the effect of diffusion of responsibility on first-order cooperation (i.e. thieves’ propensity to steal) and the effect of higher expected payoffs from stealing in larger groups. This is because *U* is constant across conditions whereas the amount that can be stolen increases with group size (*nU*). In other words, thieves’ incentives to steal increase both because of the larger amount that can be stolen *and* the lower probability of a sanction in larger groups. We chose to keep *U* rather than *nU* constant across group size because the main aim of this study is to test the effect group heterogeneity has on the diffusion of responsibility in social norm enforcement. The effect of changing sanctioning probabilities on first-order cooperation has been corroborated elsewhere [15,37]. We therefore refrain from making predictions about the effect of experimental conditions on first-order cooperation (i.e. stealing). However, in the Results section we also report our findings regarding the stealing rate.

### Predictions and hypotheses

Based on our parametrization of the VOD specified in Table 1, we can derive actors’ probabilities of enforcing the social norm (i.e. sanction), the probabilities that at least one group member will sanction and the social norm will be enforced, and the probabilities that exactly one group member will sanction and the social norm will be enforced efficiently. The point predictions are listed in the upper half of Table 2; the formal derivation of these predictions is explained in all detail elsewhere (see [15,37]).

**Table 2:** Predicted sanctioning probabilities.

	symmetric		asymmetric	
	*n* = 2	*n* = 5	*n* = 2	*n* = 5
*p*_i_*	0.500	0.159	n/a	n/a
*p*_s_*	n/a	n/a	1	1
*p*_w_*	n/a	n/a	0	0
p*	0.750	0.580	1	1
*p*_e_*	0.500	0.398	1	1

*n*: group size; *p*_i_*: equilibrium sanctioning probability; *p*_s_*: equilibrium sanctioning probability of strong player; *p*_w_*: equilibrium sanctioning probability of weak player; p*: probability that at least one sanction will occur in equilibrium; *p*_e_*: probability that exactly one sanction will occur in equilibrium.

In the symmetric VOD, actors’ sanctioning probabilities are predicted to be the same for all actors in a group and are denoted with *p*_i_*. In groups of *n* = 2, *p*_i_* = 0.500, and in groups of *n* = 5, *p*_i_* = 0.159. In other words, an actor’s probability to sanction decreases with group size, which corresponds to the diffusion of responsibility effect. The diffusion of responsibility effect is also predicted at the group level. The probability that at least one actor sanctions is p* = 0.750 in groups of *n* = 2, and it is p* = 0.580 in groups of *n* = 5. The diffusion of responsibility will also be reflected in the probability that exactly one actor sanctions and enforces the social norm efficiently, which is *p*_e_* = 0.500 in groups of *n* = 2, and *p*_e_* = 0.398 in groups of *n* = 5.

In contrast, in the asymmetric VOD, the “weak” actors are predicted to sanction with probability *p*_w_* = 0 and the strong actors sanction with probability *p*_s_* = 1, irrespective of group size. Hence, no diffusion of responsibility is predicted to occur in the asymmetric VOD, neither at the individual nor at the group level (p* = 1), and the social norm is always enforced by the strong actor alone (*p*_e_* = 1). Based on these point predictions, we can state the following hypotheses:

Symmetric VOD: n = 2 vs n = 5

H1.1: In the symmetric VOD, subjects will less often sanction in groups of *n* = 5 than in groups of *n* = 2.

H1.2: In the symmetric VOD, the social norm will less often be enforced in groups of *n* = 5 than in groups of *n* = 2.

H1.3: In the symmetric VOD, the social norm will less often be enforced *efficiently* in groups of *n* = 5 than in groups of *n* = 2.

Asymmetric VOD: weak subject vs strong subject

H2: In the asymmetric VOD, “weak” subjects will less often sanction than “strong” subjects.

Asymmetric VOD: n = 2 vs n = 5

H3.1: In the asymmetric VOD, “strong” subjects will as often sanction in groups of *n* = 5 as in groups of *n* = 2.

H3.2: In the asymmetric VOD, the social norm will as often be enforced in groups of *n* = 5 as in groups of *n* = 2.

H3.3: In the asymmetric VOD, the social norm will as often be enforced *efficiently* in groups of *n* = 5 as in groups of *n* = 2.

Symmetric VOD vs asymmetric VOD

H4.1: In the symmetric VOD, (weak) subjects will less often sanction than “strong” subjects in the asymmetric VOD.

H4.2: In the symmetric VOD, the social norm will less often be enforced than in the asymmetric VOD.

H4.3: In the symmetric VOD, the social norm will less often be enforced *efficiently* than in the asymmetric VOD.

### Experimental procedures

In total, 252 subjects participated in our computerized laboratory experiment. The experiment comprised seven sessions and 36 subjects participated in each session. Because of the random matching protocol and the large groups size in one of the treatments, we varied group size between sessions. The treatment with five victims (*N* = 5 + 1) was conducted in sessions 1, 3, 5 and 7, and the treatment with two victims (*N* = 2 + 1) was conducted in sessions 2, 4 and 6. In sessions testing the treatment with two victims, subjects were randomly assigned to the symmetric VOD (subjects 1 through 18) or asymmetric VOD (subjects 19 through 36). In the treatment with five victims the type of VOD was varied across sessions.

The experiment was conducted in the Decision Science Laboratory at ETH Zurich (DeSciL). Subjects were students from the University of Zurich and ETH Zurich, 59.5% were female and they were 22.7 years old on average (sd = 3.00). Upon arrival in the lab, subjects received condition-specific instructions on paper. The instructions that were given to subjects in one of the experimental conditions (asymmetric VOD with *n* = 5) are provided in the online Supporting Information (S1 Additional study material and analyses). Instructions explained the decision situations step by step and contained shots of the actual decision screens. Moreover, subjects learned that their decisions were anonymous, that their payments would correspond to the sum they earned in each round and that payments would be administered by a person not involved in the implementation of the experiment. After reading the instructions, subjects took a quiz with questions about the decision situations. Questions for which at least one wrong answer was given were read out loud and the correct answer was explained to all subjects at the same time. Then, the experiment started. A session lasted for about 1h and subjects earned CHF 38 (incl. CHF 10 show-up fee) on average (≈ USD 43.4). After the experiment, subjects filled in a questionnaire and could leave the lab to get their payment in private. The experiment was programmed and conducted with the software z-Tree [60].

### Data analysis strategy

All result figures and test statistics are based on regression model estimations. We only use logistic regression models because all our target variables are binary [61]. Statistical significance is set at the 5% level (i.e. α = 0.05) for two-sided tests and we account for the repeated measures obtained on the same subject by estimating cluster-robust standard errors [62,63]. We use Stata’s margins command to calculate proportions from saturated logit models and test the statistical significance of the differences between proportions using Wald tests of linear hypotheses. The data and Stata 14 script producing the results and figures reported in this paper are available as online Supporting Information (S1 Data, S1 Stata do-file).

## Ethics statement

This research was approved by the Ethics Committee of ETH Zurich. All the research was performed in the Decision Science Laboratory(DeSciL) at ETH Zurich, Haldeneggsteig 4, CH-8092 Zurich, Switzerland. The review board of DeSciL is called DeSciL Review Board, and its members are listed on the DeSciL website (https://www.descil.ethz.ch/people). Our experiment was conducted in accordance with DeSciL Operational Rules, which are approved by the review board and published on the DeSciL website (https://www.descil.ethz.ch/lab/researchers). All participants in our experiment were recruited from the subject pool maintained by the University Registration Center for Study Participants (UAST) of the University of Zurich and ETH Zurich. Every person who has signed up to this subject pool also gave his or her consent by agreeing to the terms and conditions of UAST via the online registration tool. Consent is required for registration and is given by selecting the checkbox labelled “I have read the terms and conditions and I agree with them.” These terms and conditions are published on the UAST website (https://www.uast.uzh.ch/register). Terms and conditions inform about the procedure of PC decision experiments in general, the anonymity of the data, and the usage of the anonymized data for the purpose of scientific studies.

## Results

**Fig 2:**
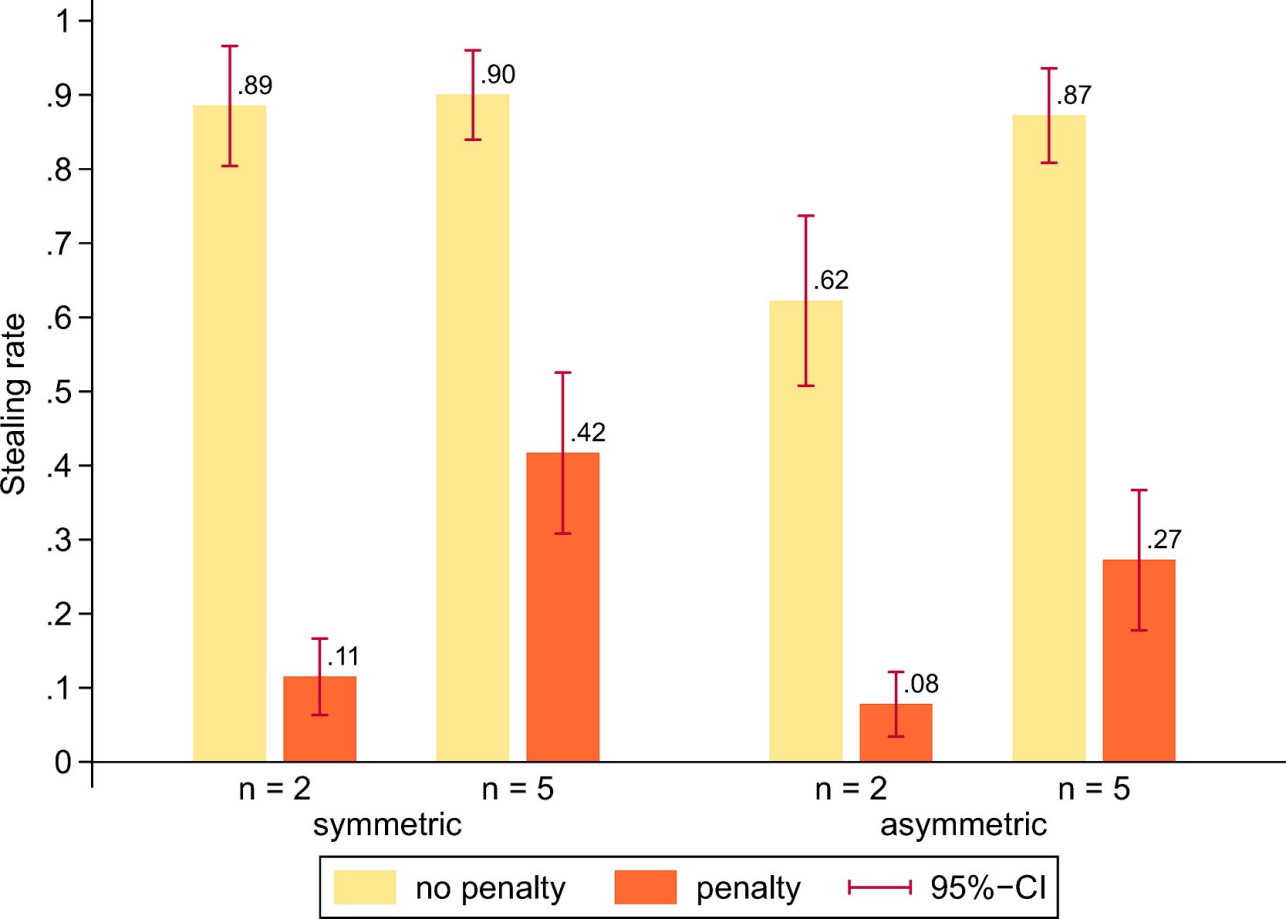
Stealing rates across experimental conditions.

Fig 2 shows stealing rates (i.e. social norm violations) across experimental conditions. Across all conditions there is a substantial and significant penalty effect. That is, the stealing rates are considerably lower in the last 15 rounds as compared to the first 15 rounds, and the overall reduction is significantly larger in groups of *n* = 2 than in groups of *n* = 5 (*z* = 3.08, *p* = 0.002). In groups of *n* = 2 there is no significant difference in the stealing rate between the symmetric and the asymmetric condition with penalty (*z* = 1.46, *p* = 0.144). In groups of *n* = 5, the stealing rate is significantly lower in the asymmetric condition than the symmetric condition (*z* = 2.92, *p* = 0.004). Without penalty (in the first 15 rounds), only the stealing rate in the asymmetric *n* = 2 condition is significantly lower than the stealing rates in the other three conditions (see Fig 2). Recall that our experimental design does not allow us to disentangle the effects of diffusion of responsibility and expected payoffs of stealing on stealing rates because both factors increase the incentive to steal as groups become larger (see Experimental design). We therefore refrain from stating and testing hypotheses relating experimental conditions to stealing behavior.

The penalty effect leads to less stealing and thus a much smaller number of observations at the sanctioning stage (Fig 2). We therefore restrict our reporting of main results to the first 15 rounds, without a penalty threat for the thief. The analysis of the second 15 rounds, with a potential penalty for the thief, yields similar results and is reported in the online Supporting Information (S1 Additional study material and analyses). In the following we first report results pertaining to individual level behaviour (i.e. sanctioning rate), then the results regarding the rate of social norm enforcement (i.e. collective good production) and lastly the results regarding the rate of *efficient* social norm enforcement.

### Testing hypotheses

**Fig 3:**
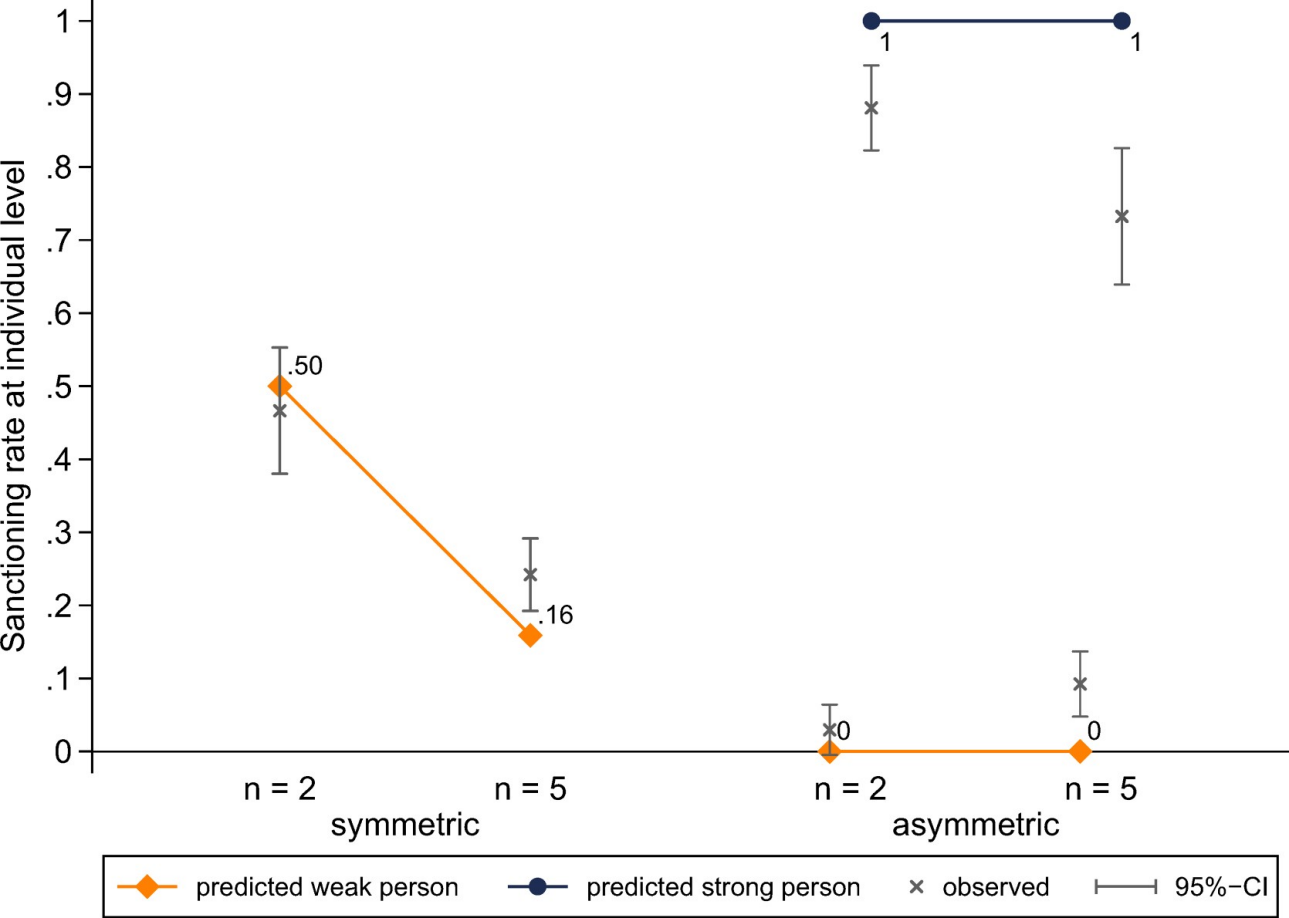
Predicted and observed sanctioning rates at the individual level across experimental conditions and subject roles.

Fig 3 shows predicted and observed individual sanctioning rates across experimental conditions and subject roles. In line with hypothesis H1.1, we observe a strong diffusion of responsibility effect in the symmetric conditions. The decrease in the individual sanctioning rate from 0.47 to 0.24 if group size increases from two to five is statistically significant (*z* = 4.42, *p* < 0.001). Moreover, we observe a substantial disparity of subjects’ behaviour between the symmetric and asymmetric VOD. In the asymmetric VOD, in line with hypotheses H2, strong subjects sanction and weak subjects refrain from sanctioning almost all the time. These results are also in line with hypothesis H4.1, according to which weak subjects in the asymmetric VOD will sanction less than (weak) subjects in the symmetric VOD (*n* = 2: *z* = 9.20, *p* < 0.001; *n* = 5: *z* = 4.39, *p* < 0.001). Subjects’ behaviour is thus very much in accordance with the asymmetric equilibrium predictions [35,36,38]. As expected, the decrease in the individual sanctioning rate across group size is smaller in the asymmetric dilemma (from 0.88 to 0.73), but the difference is still significant (*z* = 2.65, *p* = 0.008). Moreover, the difference in differences is statistically insignificant (*z* = 1.01, *p* = 0.315). These result go clearly against our hypothesis H3.1. We will come back to this point shortly.

**Fig 4:**
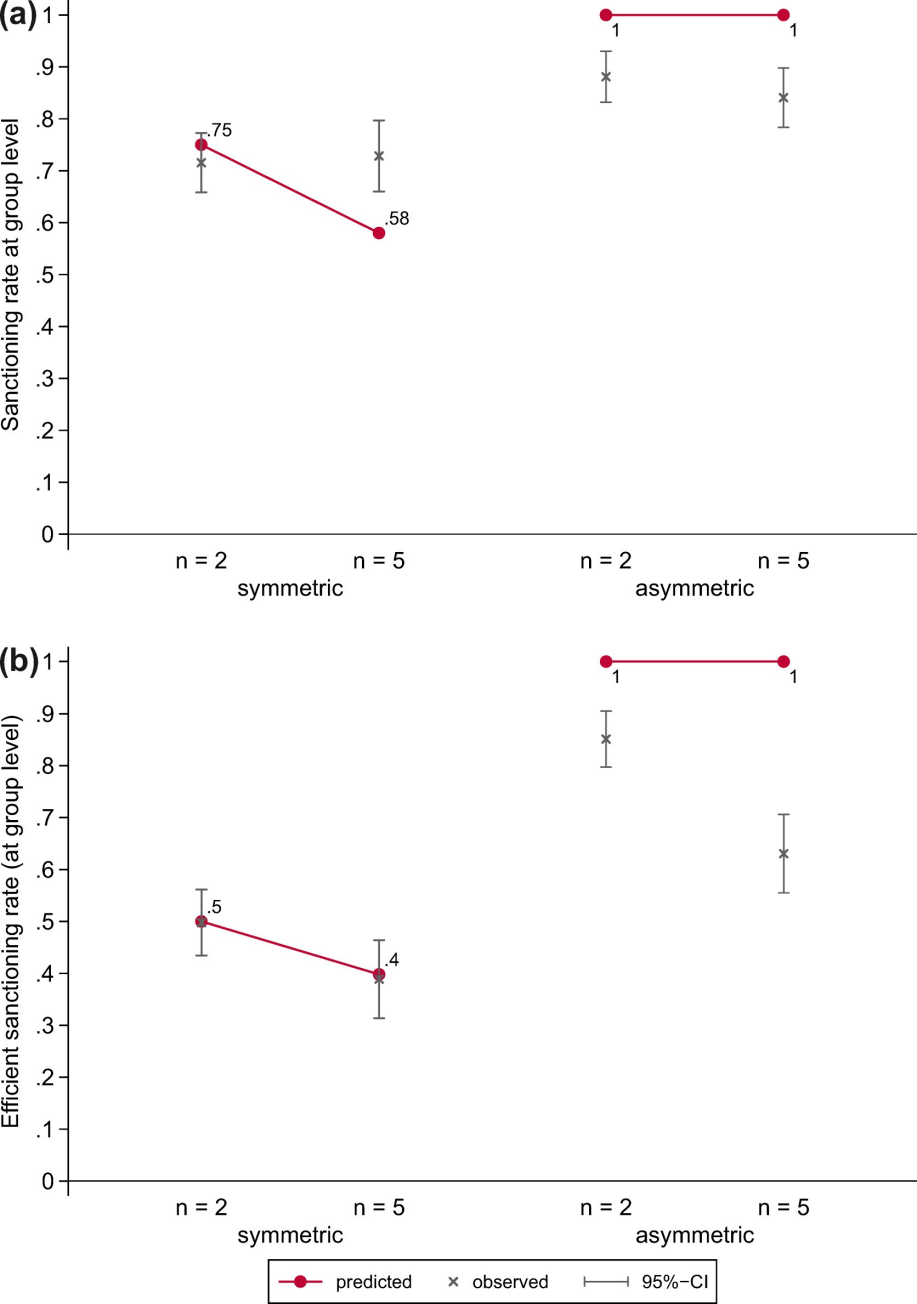
Predicted and observed sanctioning rates at the group level across experimental conditions. (a) Sanction by at least one person. (b) Sanction by exactly one person].

Fig 4A shows the predicted and observed sanctioning rates at the group level across experimental conditions. The sanctioning rate at the group level denotes the proportion of cases in which *at least one* victim sanctioned the thief. We find no support for hypothesis H1.2; there is no statistically significant decrease in the sanctioning rate when group size increases from *n* = 2 to *n* = 5 in the symmetric VOD (*z* = -0.28, *p* = 0.777). That is, unlike at the individual level, there is no evidence for a decrease of collective good production at the group level. At the same time, we cannot reject hypothesis H3.2; there is no significant difference between sanctioning rates in groups of size *n* = 2 and *n* = 5 in the asymmetric VOD (*z* = 1.04, *p* = 0. 296). We find clear support for hypothesis H4.2; the social norm is less often enforced in the symmetric than in the asymmetric VOD both in groups of *n* = 2 (*z* = -4.30, *p* < 0.001) and in groups of *n* = 5 (*z* = -2.47, *p* = 0.014).

The efficient sanctioning rate (both predicted and observed) presented in Fig 4B denotes the proportion of cases in which *exactly one* victim sanctioned the thief. In line with the diffusion of responsibility hypothesis (H1.3), the figure shows that group size has a negative effect on the efficient sanctioning rate in the symmetric VOD (*z* = 2.17, *p* = 0.030). However, a significant decrease in the efficient sanctioning rate with group size can also be observed in the asymmetric VOD (*z* = 4.66, *p* < 0.001), which leads us to reject hypothesis H3.3. Still, we find clear support for our hypothesis (H4.3) that the second order public good is less often produced efficiently in the symmetric VOD than in the asymmetric VOD both in groups of *n* = 2 (*z* = -8.32, *p* < 0.001) and in groups of *n* = 5 (*z* = -4.45, *p* < 0.001).

Out of our ten hypotheses, seven are supported and three are rejected by the empirical evidence. The three rejected hypotheses are on the diffusion of responsibility effect (three out of six), one in the symmetric VOD predicting diffusion of responsibility to occur with an increase in group size (H1.2) and two in the asymmetric VOD predicting the absence of diffusion of responsibility (H3.1 and H3.3). We have good reason to assume that this is not a stable result. In what follows, we formulate our conjectures and perform exploratory analyses using the same data. However, the findings resulting from this exploratory analysis can only be interpreted as first evidence and must be verified in further experiments.

### Exploratory analyses

Our conjecture is that in the asymmetric VOD subjects take longer to tacitly coordinate on the strong subject to sanction the thief alone if groups are larger. In the asymmetric VOD with two players, the difference in payoffs becomes immediately apparent; in the asymmetric VOD with five players, it might take a while for subjects to realize that one of them has lower costs for sanctioning (despite the fact that this is made clear in the instructions). Hence, over time, subjects will learn to appreciate the strategic nature of the decision situation and tacitly coordinate on the strong subject to sanction alone. Although we did not *a priori* hypothesize a “learning effect” (in fact an interaction between learning and group size in the asymmetric VOD), we can control for time in a more detailed, exploratory analysis.

We divide the 15 rounds without penalty (see Table 1) in blocks of five rounds (i.e. rounds 1 to 5, 6 to 10, and 11 to 15) and perform additional analyses to test our conjecture. The division in three blocks of five rounds is a natural choice because with fewer blocks we may not be able to capture the time trend implicit in our conjecture and with more blocks (e.g. five blocks of three rounds) the low number of cases in the last block will decrease the power of the statistical tests of our conjecture. We did not perform any additional analysis based on other divisions of the fifteen rounds.

**Fig 5:**
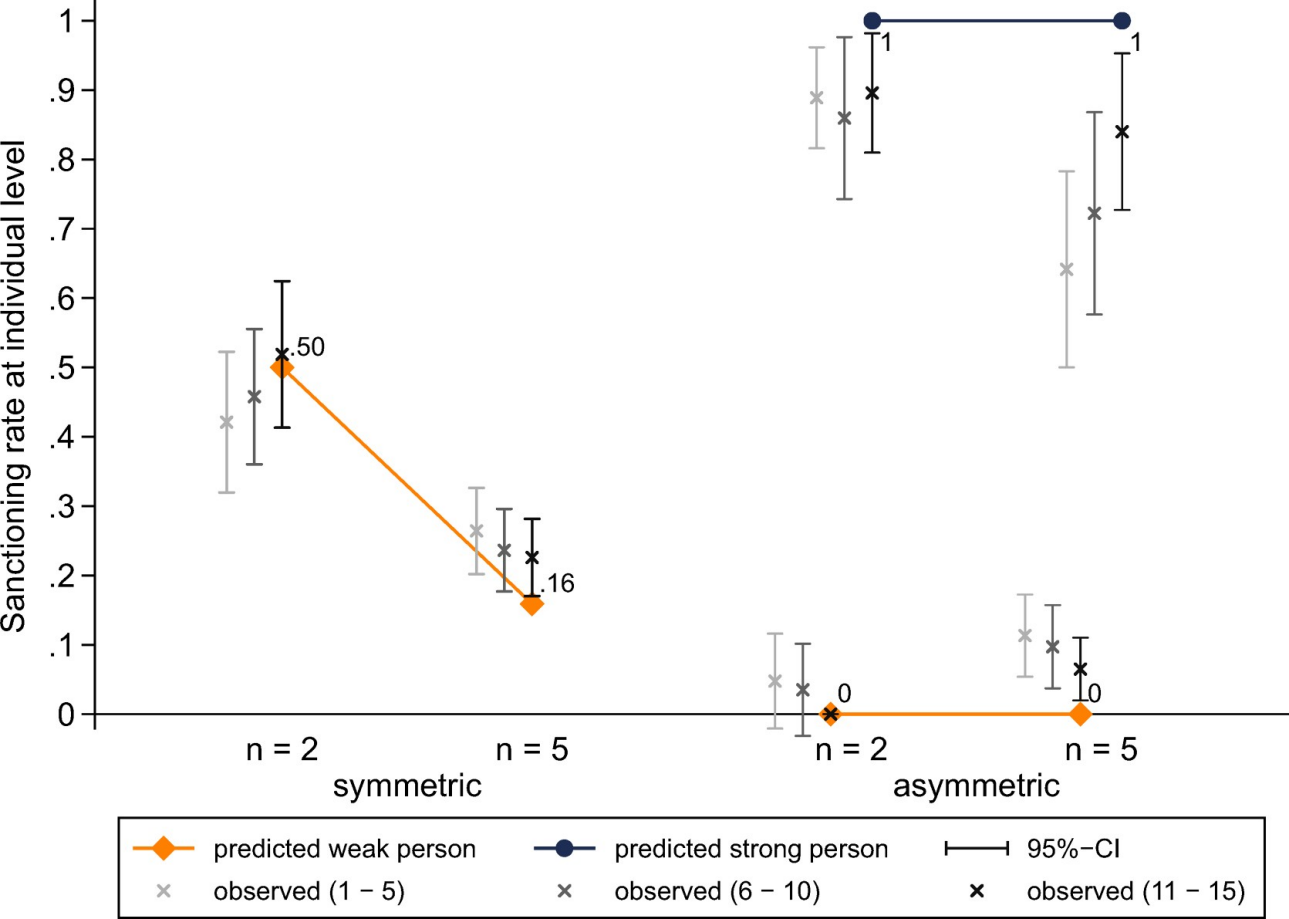
Predicted and observed sanctioning rates at the individual level across experimental conditions, subject roles and time.

In the same way as Fig 3, Fig 5 shows predicted and observed individual sanctioning rates across experimental conditions and subject roles. But unlike in Fig 3, the observed rates are also shown over time. In support of our conjecture, Fig 5 shows that the sanctioning rate of the strong subject increases over time in the *n* = 5 condition. As a result, in the last five rounds (i.e. rounds 11 to 15), the group size effect in the asymmetric VOD is not significant anymore (*z* = 0.77, *p* = 0.441). Hence, subjects seem to take longer to adjust their behaviour in the more complex decision task with five group members. Now we also find that, in the last five rounds, the difference in differences between the symmetric and asymmetric VOD is statistically significant (0.90–0.84 vs 0.52–0.23; *z* = 2.50, *p* = 0.012). That is, if group size increases from two to five, the decrease in the strong player’s sanctioning rate is significantly smaller than the decrease in the individual sanctioning rate in the symmetric VOD.

**Fig 6:**
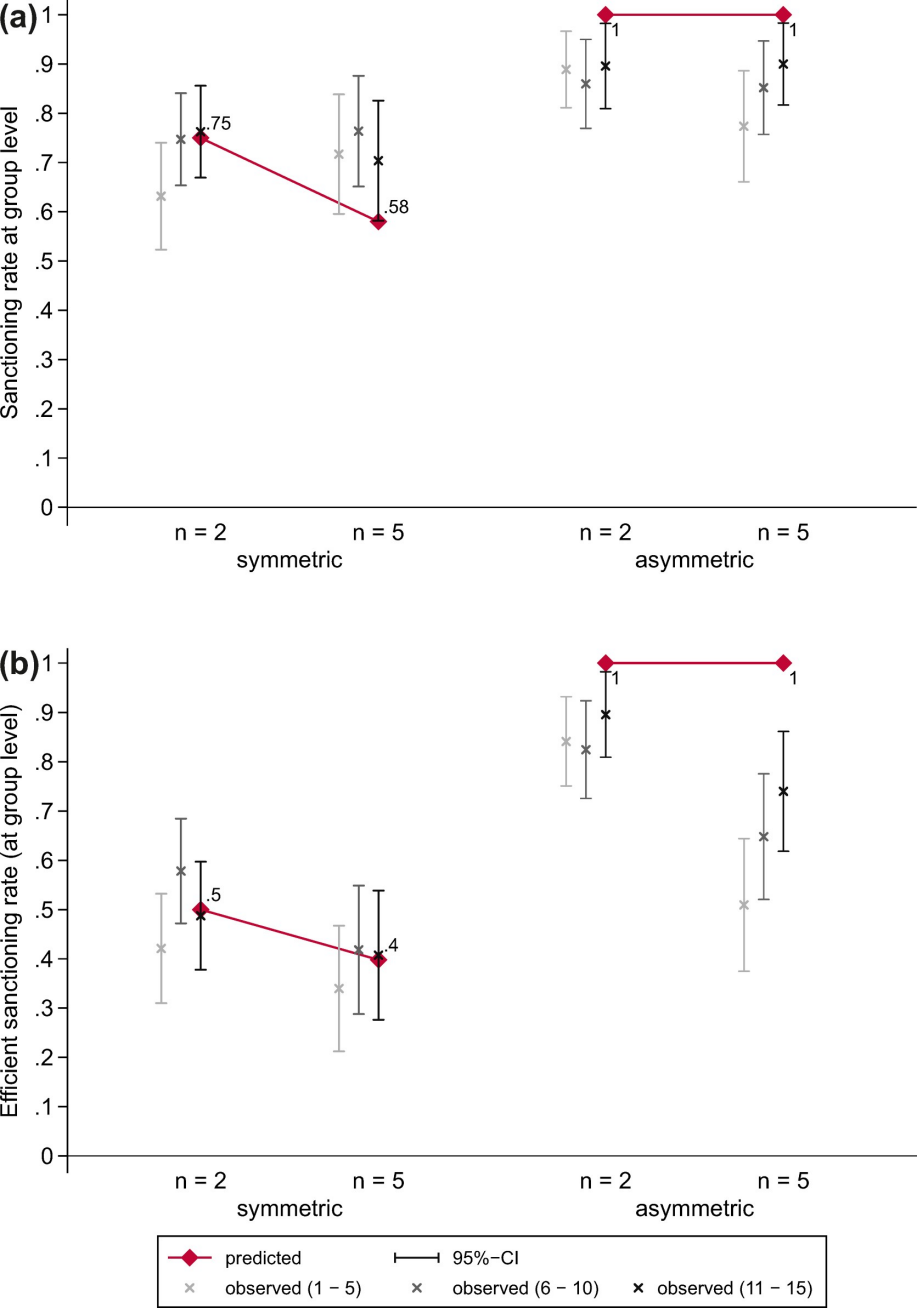
Predicted and observed sanctioning rates at the group level across experimental conditions and time. (a) Sanction by at least one person. (b) Sanction by exactly one person.

Fig 6A shows the predicted and observed group-level sanctioning rates (where the sanction was performed by at least one subject) across experimental conditions and time. Although the diffusion of responsibility effect in the symmetric VOD remains absent (i.e. H1.2 remains unsupported), the figure shows that in the asymmetric VOD with *n* = 5 subjects’ behavior converges to the predicted sanctioning rate. Fig 6B shows the predicted and observed efficient sanctioning rates (where the sanction was performed by exactly one subject) across experimental conditions and time. Although there is a statistically significant increase in the efficient sanctioning rate in the asymmetric VOD with *n* = 5 from the first five to the last five rounds (*z* = 2.49, *p* = 0.013), the sanctioning rate in the last five rounds remains significantly lower than the rate in the asymmetric VOD with *n* = 2 (*z* = 2.05, *p* = 0.041). All in all our exploratory analyses corroborate our conjecture that subjects need more time to reach the sanctioning rates predicted in the asymmetric VOD if groups are larger.

## Discussion

Five decades ago, Darley and Latané [19] showed that an emergency situation may end tragically despite the presence of potential helpers if each of them expects someone else to intervene. The authors called this mechanism “diffusion of responsibility.” Their recognizing the mechanism and corroborating it empirically was an important step towards a better understanding of the dynamics and outcomes of emergency situations. Although the observation of diffusion of responsibility in different types of volunteering situations is very robust [20,30], there are also exceptions. Here we argue that observable asymmetries of the bystanders in terms of their costs of and/or benefits from intervening may account for these exceptions (see also [47,64,65]). To test this conjecture, we model the volunteering situation as a symmetric and asymmetric volunteer’s dilemma game (VOD), derive hypotheses about the moderating effect of asymmetry on the diffusion of responsibility in social norm enforcement, and test our predictions in a computerized lab experiment.

In our experiment, we use the stealing game with a sanctioning option to emulate a situation in which a norm violation can be negatively sanctioned. That is, in a group of subjects, one randomly chosen subject can decide to steal money from the other group members (i.e. violate a social norm), who then face a VOD as only one of them is required to reclaim the stolen amount for the entire group (i.e. enforce the social norm). We vary group size and symmetry of the VOD in terms of the costs a subject incurs from sanctioning the thief. Our results show a clear diffusion of responsibility effect in the symmetric VOD, in which all group members have the same costs of sanctioning the thief. In the asymmetric VOD, diffusion of responsibility is largely diminished but only after subject have played the game for some time. By and large, these results are also borne out at the group level. In particular, heterogeneous groups become more effective in enforcing social norms as they manage to tacitly coordinate on the strongest subject to sanction the norm breaker alone. Our findings support the proposition that even relatively small asymmetries in observable sanctioning costs facilitate bystanders’ tacit coordination on the “strongest” individual to negatively sanction norm breakers. In other words, our results show how asymmetry can “break” the diffusion of responsibility in social norm enforcement and help to overcome the second-order free-rider problem [53,66,67].

Our findings complement results from field experiments in social psychology in which the perception of competent bystanders inhibits cooperation of other group members [55,68]. There is also supporting evidence from research on coordination behaviour in non-human primates and small children [69,70]. In groups of chimpanzees the group member with highest rank takes solitary action in a volunteer’s dilemma [18]. Because the chimpanzee’s rank is negatively correlated with the costs of cooperation, this experimental observation is in line with our results on the asymmetric VOD. Asymmetry can also establish a focal point for individuals to tacitly coordinate on a unique equilibrium in a situation with multiple equilibria [15,71,72]. Such an ability to tacitly coordinate on a focal equilibrium seems to emerge in children as from the age of five [73].

In how far asymmetry in costs from intervening provides an incentive for, and in how far it merely establishes a focal point facilitating coordination on the “strongest” individual to volunteer in the asymmetric VOD is an open question to be addressed in future research. Future research should also reconsider the effect of individual heterogeneity on collective action and cooperation in groups. Although predicted by theory [35,36], it is an empirical question whether introducing payoff differences in weak subjects as well (while keeping one identifiable strong subject) would produce similar results as reported above. Our conjecture is that a gradual distinction of subjects strength in terms of payoffs (costs and or benefits of intervening) would also facilitate tacit coordination on the strongest subset of *k* > 1 subjects that is required to produce a collective good for a group of size *n > k*. This would extend the applicability of our findings beyond the volunteer’s dilemma, for which *k* = 1.

## Supporting information

S1 Additional study material and analyses (PDF)

S1 Data (DTA)

S1 Stata do file (DO)
