# Supplementary material for: Heterogeneous groups overcome the diffusion of responsibility problem in social norm enforcement
Source: PLoS One. 2018 Nov 30;13(11):e0208129. doi: 10.1371/journal.pone.0208129 (PMC6267992; doi:10.1371/journal.pone.0208129)
Supplement: S1 Additional study material and analyses — (PDF) [file pone.0208129.s001.pdf]

Online Supporting Information for:

Heterogeneous Groups Overcome the Diffusion of Responsibility Problem in Social Norm Enforcement

Wojtek Przepiorka[1] and Andreas Diekmann[2]

[1] Department of Sociology/ICS, Utrecht University, Netherlands, w.przepiorka@uu.nl

[2] ETH Zurich, Switzerland, diekmann@soz.gess.ethz.ch

S1. Evolutionary stable equilibria in the asymmetric VOD

He et al. (2014) have shown by means of analyses of replicator dynamics and simulations that the asymmetric VOD with one strong player hast two evolutionary stable equilibria (ESE). Figure S1 illustrates the main result of their analyses. In one ESE (sink point A in Figure S1), the strong player always cooperates and the weak players never cooperate. In the second ESE (sink point E in Figure S1), the strong player never cooperates and the weak players cooperate with probability $\boldsymbol{p_w^* = 1 - \sqrt[n-2]{K_w/U_w}}$. In other words, in the second ESE, the weak players ignore the strong player and behave in accordance with the mixed-strategy equilibrium of the symmetric VOD with $n - 1$ weak players.

Note that the saddle point F in Figure S1 corresponds to the mixed strategy equilibrium of the asymmetric VOD. Like the other saddle point C and the source points B and D, F does not constitute a stable equilibrium state of the game. Moreover, the first ESE (A) corresponds to the equilibrium selected by rationality theory and also has a larger basin of attraction than the second ESE (E) (He et al., 2014). We therefore base our predictions for the asymmetric VOD on the first ESE, in which the strong actor always cooperates while all weak actors abstain from cooperation.

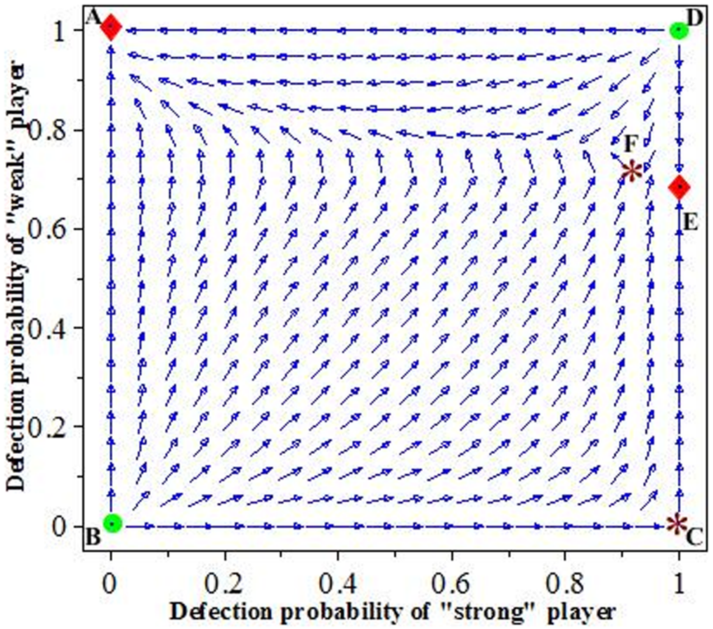

Fig S1. The slope field for replicator dynamics of the asymmetric volunteer's dilemma (He et al. 2014).

S2. Experimental instructions (translated from German by the authors)

Introduction

Please read these instructions attentively.

The experiment will last for about 60 minutes, it consists of two parts and each part comprises 15 rounds. You are receiving the instructions concerning the first part of the experiment on these three pages, which you may use as a reminder during the experiment. Instructions concerning the second part are short and you will receive them at the end of the first part on your screen only. Let us start with some important information.

You are participating in an experiment in which you will earn some money. The amounts you earn in each of the 30 rounds will be summed up at the end, and you will be paid this amount together with your show-up fee of 10 sFr. in cash. The payment will be made by an assistant that was not involved in the implementation of this experiment. Your earnings depend on both the decisions you will make and the decisions other participants will make. There are no right or wrong decisions and you make all your decisions anonymously.

All participants are receiving the same instructions and take part in this experiment under the same conditions. Please, from now on, do not talk to each other anymore and switch off your mobile devices. This experiment is being conducted by the Chair of Sociology at ETH Zurich and the data collected in this experiment are for scientific purposes only.

1

Figure S2: Instructions (asymmetric VOD, *n* = 5; page 1)

Instructions (Part 1)

The first part of the experiment will last for 15 rounds. In each round you are with five different people in a group and a group consists of a *Person 1*, a *Person 2*, a *Person 3*, a *Person 4*, a *Person 5* and a *Person X*. That is, at the beginning of each round, the groups are formed randomly anew (that is, in each round you will be with different participants in a group) and it is determined by chance whether you are *Person 1*, *Person 2*, *Person 3*, *Person 4*, *Person 5* or *Person X*.

In each round, each participant receives an endowment of 100 Rp. (= 1 sFr.). *Person X* (and only *Person X*) can then decide whether or not he or she would like to deduct part of the other three persons' endowments (see Figure 1). *Person X* can deduct either 0 Rp. or 50 Rp. from each of the other persons' endowments (i.e. 0 Rp. or 5 x 50 Rp. = 250 Rp.) and this amount will be added to *Person X*'s account. That is, if *Person X* decides to deduct 50 Rp. from each of the other three persons, then *Person X*'s account will amount to 350 Rp. (100 Rp. + 250 Rp. = 350 Rp.) and the other five persons' accounts will amount to 50 Rp. each (100 Rp. – 50 Rp. = 50 Rp.). If *Person X* decides to deduct 0 Rp. from the other five persons, then all six persons will keep their endowment of 100 Rp.

Figure 1: Decision of Person X

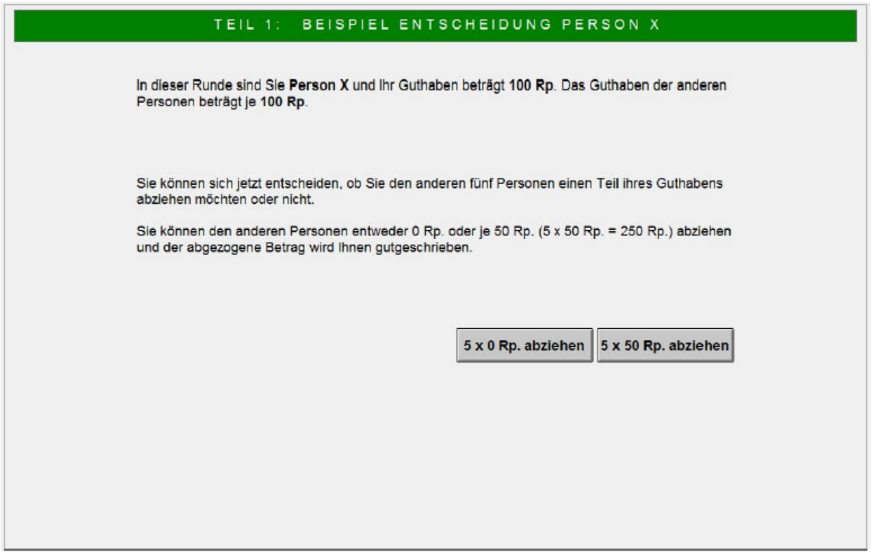

2

Figure S3: Instructions (asymmetric VOD, n = 5; page 2)

If *Person X* decides to deduct 50 Rp. from each of the other five persons, then these five persons can decide independently whether or not they want to reclaim this amount from *Person X*. If *Person X* decides not to deduct anything from the other five persons, then, of course, these five persons do not have the possibility to reclaim anything from *Person X*.

Hence, if *Person X* decides to deduct 50 Rp. from each of the other five persons, the decision situation depicted in Figure 2 appears on the other five persons' screens. Then, the five persons decide independently whether they want to choose "up" or "down" in their decision field. If at least one of the three chooses "up", the account of *Person X* will be reduced back to 100 Rp. (350 Rp. – 250 Rp. = 100 Rp.) and the other five persons' accounts will amount to 100 Rp. each again (50 Rp. + 50 Rp. = 100 Rp.). However, *Person 2* will be charged 25 Rp. if he or she chooses "up". *Persons 1, 3, 4* and *5* will be charged 35 Rp. for choosing "up". A person that chooses "down" will not be charged anything. If, however, all five persons choose "down", the amount that *Person X* deducted from their accounts will not be reclaimed.

Figure 2: Decision of Person 1, 2, 3, 4 and 5

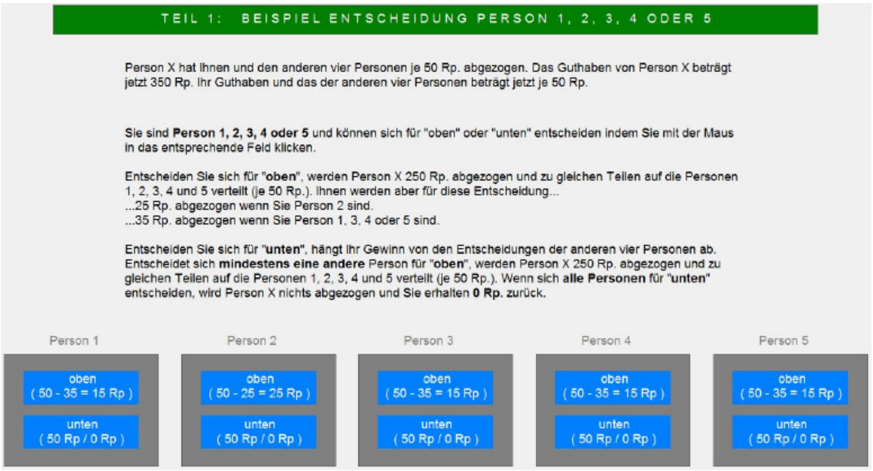

Before the next round, the groups of four will be disbanded and formed randomly anew, and it will be determined by chance whether you are *Person 1*, *Person 2*, *Person 3*, *Person 4*, *Person 5* or *Person X*. It could therefore be that you will never be Person X, for instance, or only at irregular intervals.

3

Figure S4: Instructions (asymmetric VOD, $n = 5$; page 3)

Instructions (Part 2) [on screen only, after Part 1]

The second part of the experiment will last for 15 rounds and differs from the first part of the experiment as follows:

If *Person X* decides to deduct 50 Rp. from each of the other five persons and then at least one of the other five persons chooses "up", the account of *Person X* will be reduced by an additional 60 Rp. to 40 Rp. instead of 100 Rp. as before. *Person X*'s account will always be reduced at most by an additional 60 Rp., irrespective of whether one, two, three, four or all five of the other persons choose "up". If all five persons choose "down", the amount that *Person X* deducted from their accounts will not be reclaimed and the account of *Person X* will not be additionally reduced. Everything else stays the same.

4

Figure S5: Instructions (asymmetric VOD, n = 5; page 4)

S3. Further data analyses and results

Figure S6 shows the individual (a), group (b) and efficient group veto rate (c) across treatments in the last 15 rounds (with a penalty threat for thieves). Except for the group veto rate, the results shown in Figure S6 are in line with the results obtained in the first 15 rounds (without a penalty threat for thieves). As in the first 15 rounds, there is a significant decrease in the individual veto rate (panel a) in the symmetric VOD ($z = 4.05$, $p < 0.001$) but not in the asymmetric VOD ($z = 1.57$, $p = 0.117$) if group size increases from $n = 2$ to $n = 5$. The difference in differences is, however, insignificant ($z = 1.52$, $p = 0.129$). Unlike in the first 15 rounds, the group veto rate (panel b) does not differ between the symmetric and asymmetric VOD neither in groups of $n = 2$ ($z = -0.72$, $p = 0.474$) nor in groups of $n = 5$ ($z = -0.06$, $p = 0.953$). However, for the efficient group veto rate (panel c) both these differences are statistically significant ($z = -2.93$, $p = 0.003$ and $z = -2.50$, $p = 0.012$, respectively).

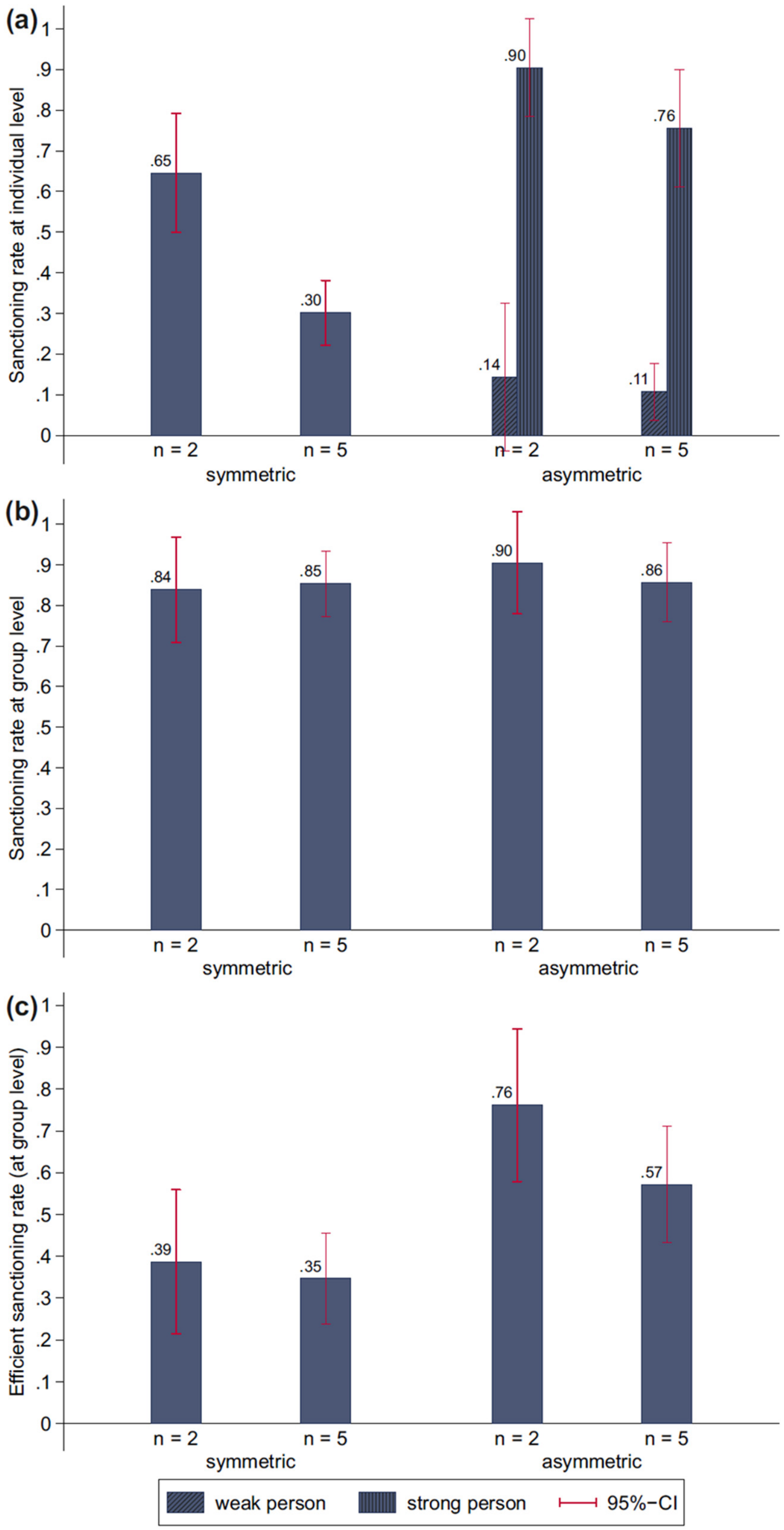

Fig S6. Individual (a), group (b) and efficient group sanctioning rate (c) across treatments in the last 15 rounds (with a penalty threat for thieves).
